# Nuclear transport maintenance of USP22-AR by Importin-7 promotes breast cancer progression

**DOI:** 10.1038/s41420-023-01525-8

**Published:** 2023-07-01

**Authors:** Geng-Xi Cai, Wei-Yao Kong, Yuan Liu, Shu-Yi Zhong, Qing Liu, Yuan-Fei Deng, Guo-Lin Ye

**Affiliations:** 1 Department of Breast Surgery, The First People’s Hospital of Foshan, 528000 Foshan, Guangdong China; 2 Guangzhou Municipal and Guangdong Provincial Key Laboratory of Protein Modification and Degradation, School of Basic Medical Sciences, Guangzhou Medical University, 511436 Guangzhou, Guangdong China; 3 Guangzhou Medical University-Guangzhou Institute of Biomedicine and Health (GMU-GIBH) Joint School of Life Sciences, Guangzhou Medical University, 511436 Guangzhou, Guangdong China; 4 Department of Pathology, The First People’s Hospital of Foshan, 528000 Foshan, Guangdong China

**Keywords:** Breast cancer, Preclinical research

## Abstract

The translocation of biological macromolecules between cytoplasm and nucleus is of great significance to maintain various life processes in both normal and cancer cells. Disturbance of transport function likely leads to an unbalanced state between tumor suppressors and tumor-promoting factors. In this study, based on the unbiased analysis of protein expression differences with a mass spectrometer between human breast malignant tumors and benign hyperplastic tissues, we identified that Importin-7, a nuclear transport factor, is highly expressed in breast cancer (BC) and predicts poor outcomes. Further studies showed that Importin-7 promotes cell cycle progression and proliferation. Mechanistically, through co-immunoprecipitation, immunofluorescence, and nuclear–cytoplasmic protein separation experiments, we discovered that AR and USP22 can bind to Importin-7 as cargoes to promote BC progression. In addition, this study provides a rationale for a therapeutic strategy to restream the malignant progression of AR-positive BC by inhibiting the high expression state of Importin-7. Moreover, the knockdown of Importin-7 increased the responsiveness of BC cells to the AR signaling inhibitor, enzalutamide, suggesting that targeting Importin-7 may be a potential therapeutic strategy.

## Introduction

The latest global cancer statistics show that the incidence of breast cancer (BC) has become the most prevalent carcinoma worldwide in 2020. The mortality of BC also ranks first among female-related cancers [1]. Clinically, BC can be mainly divided into four different molecular subtypes according to the status of estrogen receptor (ER), progesterone receptor (PR), and HER2, including luminal A, luminal B, HER2-enriched, and basal-like [2, 3]. Various anticancer strategies, including surgical treatment, traditional chemotherapies, endocrine therapies targeting ER, and HER2-targeted therapies, have achieved great benefits in many patients. However, patients with medication resistance and those who lack certain characterized targets, such as triple-negative BC (TNBC), have limited therapy alternatives. Developing novel therapeutic targets and strategies is therefore urgent for current science.

Transportation of key proteins between the cytoplasm and nucleus is critical to mammalian cells to regulate various life processes [4–6]. Dysregulation of transport function may be multifaceted, such as carcinogenesis, inflammatory responses, cell cycle transition, and apoptosis [7, 8]. Nuclear pore complex (NPC) and various types of transport receptors are characterized as key players in nucleocytoplasmic transport events. Transport receptors specifically recognize macromolecular substrates such as RNA or proteins to form specific complexes and facilitate their passage through nuclear pores [4–6, 9]. Importin-7, a transport receptor protein that belongs to the importin-β superfamily, is crucial for the import of its substrate proteins into the nucleus [10, 11]. Importin-7 has been reported to link to poor prognosis due to the substantially high expression in various malignancies, including pancreatic cancer, colorectal cancer, cervical cancer, etc. [12–14]. Nonetheless, the precise contribution of Importin-7 to BC development is unclear.

Despite the conventional view that BC is mainly estrogen-dependent and ER-driven, the existence of androgen receptor (AR) in BC is also a common phenomenon, especially in ER-negative BC [15–17]. Although the expression and prognosis of AR remain controversial in BC, most reports support that AR antagonists may play a role in ER-negative/AR-positive tumors. [16, 18–21]. The protein stability and functional activity of AR are regulated by the deubiquitinating enzyme USP22 [22–24]. Here, our study demonstrated that Importin-7 promotes the progression of BC by controlling the nuclear transport of AR and its maintainer USP22.

## Results

### Importin-7 is overexpressed and predicts poor outcomes in BC

**Fig. 1:**
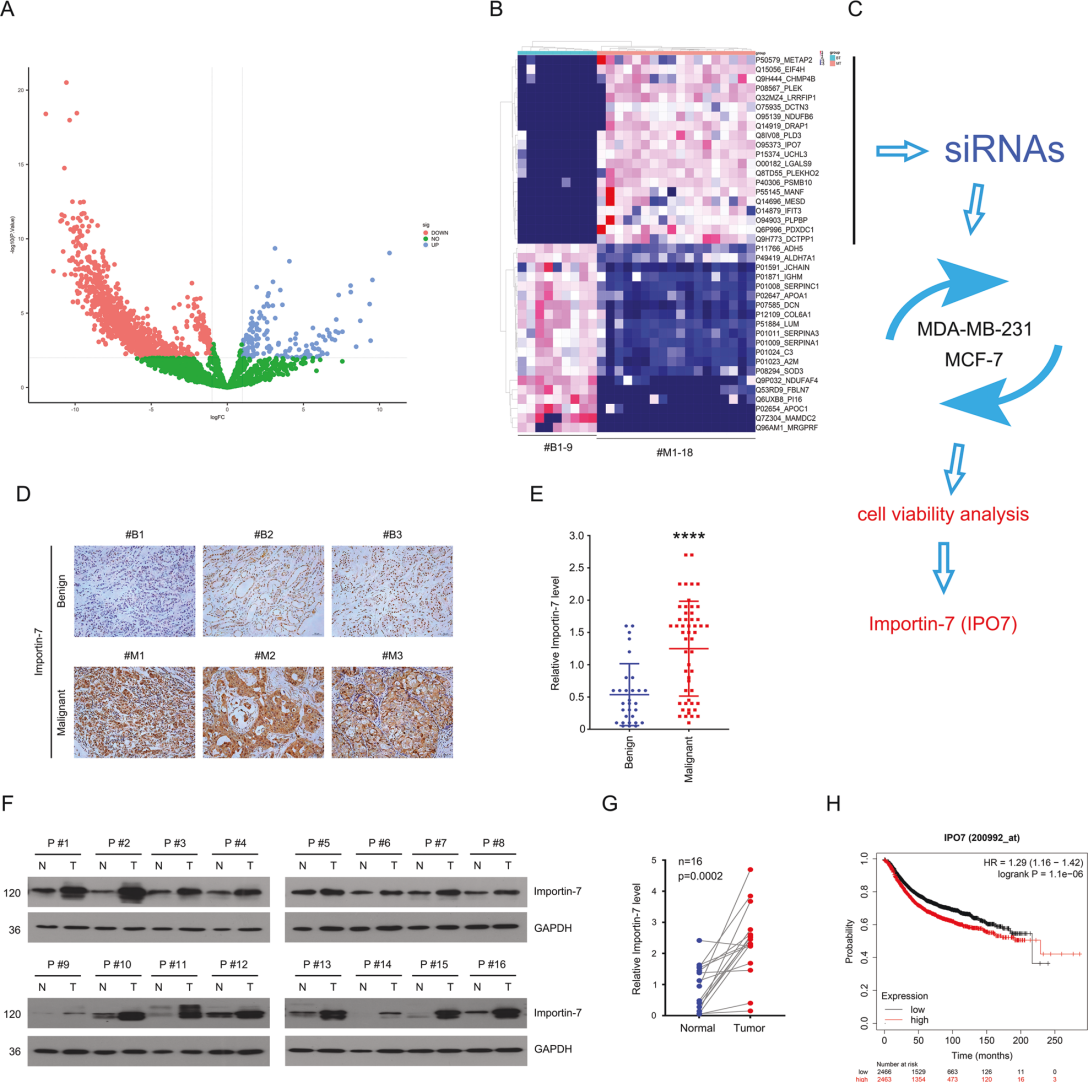
Overexpression status of Importin-7 is observed in BC. **A** Volcano plot from LC-MS/MS analysis in DIA mode in 9 cases of benign breast hyperplasia tissues and 18 cases of malignant BC tissues. **B** Heatmap for candidate proteins with a significantly different expression. **C** Schematic representation of knockdown of candidate gene expression using siRNAs in BC cells and screening by CCK8 assay. **D** IHC assay of Importin-7 on the collected 30 cases of benign breast hyperplasia and 52 cases of malignant BC. Representative images per group are shown at 200*. **E** Quantitative data of **D** are shown. **F** Western blot of Importin-7 on the adjacent normal tissues and cancer tissues of 16 BC patients. **G** Quantitative data of **F** are shown. **H** Kaplan–Meier curves from patients with BC expressing low and high Importin-7 from the TCGA database. ***p < 0.001, ****p < 0.0001 vs. each vehicle control.

With the informed consent of patients, we first detected the difference in protein levels in 9 cases of benign breast hyperplasia tissue and 18 cases of malignant BC tissue by LC–MS/MS analysis in DIA mode, the volcano plot from which showed all different protein expressions (Fig. 1A). In addition, the heatmap showed top 20 downregulated/upregulated proteins (Fig. 1B). To further explore the potential proteins that may regulate BC growth, we used small interfering RNAs (siRNAs) to knockdown the expression of the preliminarily screened genes that were notably upregulated in cancer and then detected their effects on cell viability. As one of the candidates, the knockdown of Importin-7 showed a significant tumor-suppressing effect (Fig. 1C).

Next, in order to verify the expression of Importin-7 in BC, we performed immunohistochemical detection on the collected 30 cases of benign breast hyperplasia and 52 cases of malignant BC (Fig. 1D, E). In addition, we also performed western blot detection on the adjacent normal tissues and cancer tissues of 16 BC patients (Fig. 1F, G). The results consistently showed that Importin-7 was presented in an overexpressed state in BC. Furthermore, patients with higher Importin-7 levels displayed a lower overall survival rate by analyzing the TCGA database, which suggests that the high expression status of Importin-7 indicates a poor prognosis (Fig. 1H). Therefore, it may be reasonable to suspect that Importin-7 is essential for the development of BC.

### Silence of Importin-7 suppresses the proliferation of BC cells

**Fig. 2:**
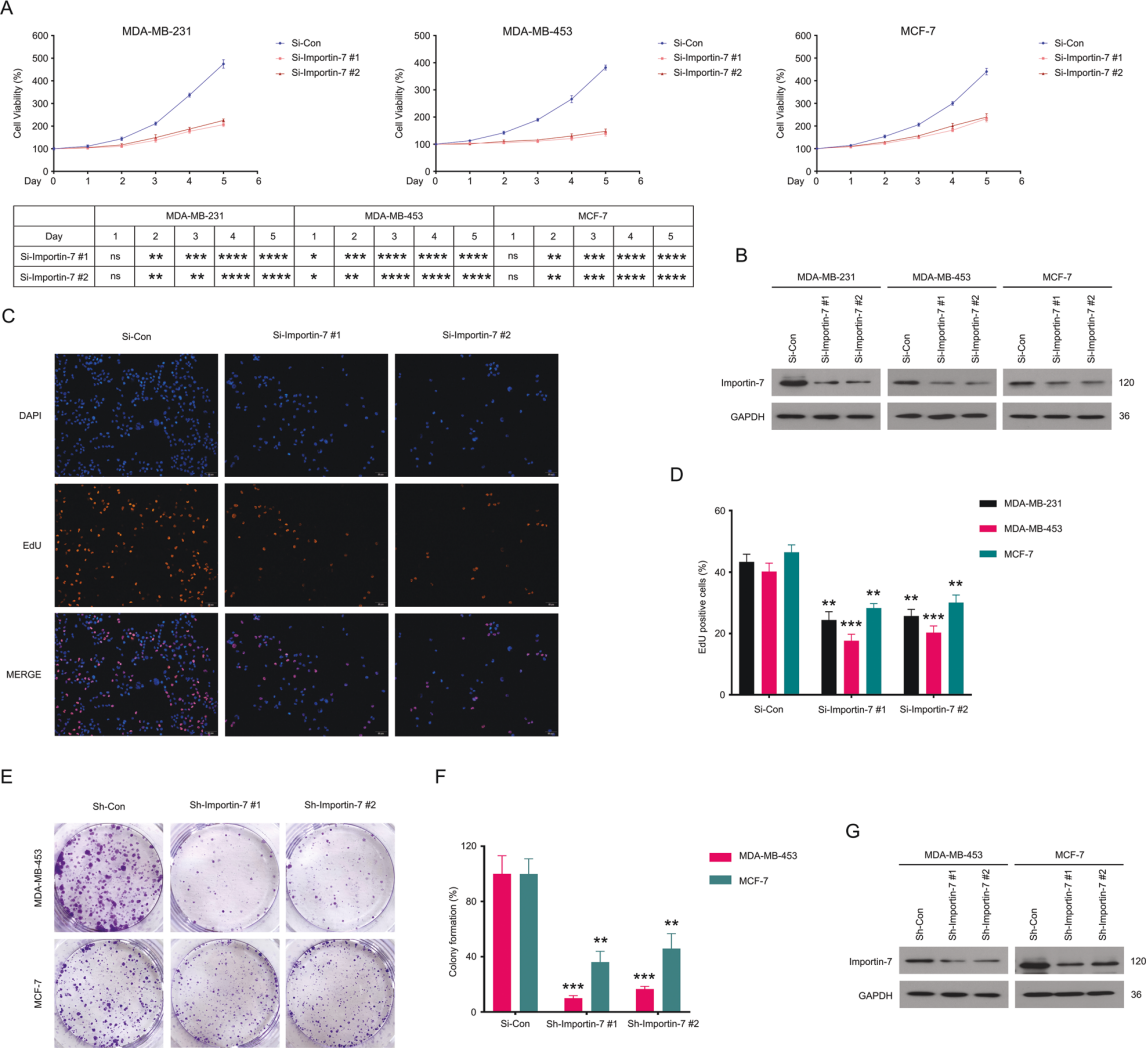
Restricting the expression level of Importin-7 suppresses the proliferation of BC cells. **A** CCK8 assays of MDA-MB-231, MDA-MB-453, and MCF-7 cells transfected with two pairs of Importin-7 or control siRNAs for 0-5 days. Statistical analyses are combined and displayed in the table below. **B** Western blot of Importin-7 in MDA-MB-231, MDA-MB-453, and MCF-7 cells transfected with Importin-7 or control siRNAs for 48 h. **C** EdU staining assay was performed in MDA-MB-231, MDA-MB-453, and MCF-7 cells transfected with Importin-7 or control siRNAs for 72 h. **D** Quantitative data of **C** are shown. Mean ± SD (*n* = 3). **E** Colony formation assay was performed in MDA-MB-453 and MCF-7 cells stably expressing control shRNA or Importin-7 shRNA for 2 weeks. **F** Quantitative data of **E** are shown. Mean ± SD (*n* = 3). **G** Western blot of Importin-7 in MDA-MB-453 and MCF-7 cells stably expressing control shRNA or Importin-7 shRNA. *p < 0.05, **p < 0.01, ***p < 0.001, ****p < 0.0001 vs. each vehicle control.

We next investigated the impact of Importin-7 knockdown on proliferation in three BC cell lines to further reveal the biological function of Importin-7 on growth. First, we used siRNAs to knock down the expression of Importin-7 in these cell lines and then used the CCK8 assay kit to detect the trend of cell viability within five days after transfection. The results showed that, from the second day of transfection, restricting the expression of Importin-7 caused significant inhibition of viability in BC cells (Fig. 2A). The knockdown efficiency of Importin-7 in the three cell lines was verified by immunoblotting experiments by means of protein expression level analysis (Fig. 2B). In addition, 72 h after transfection of Importin-7 siRNAs, the EdU staining assay was performed on the three BC cell lines to detect the DNA replication. The presented data reveal that Importin-7 knockdown notably suppressed the proliferation ability of BC cells (Fig. 2C, D).

Moreover, we established Importin-7 stable knockdown BC cell lines by transfecting lentiviruses containing two pair Importin-7 shRNAs and further performed clone formation experiments on these BC cells. We showed that Importin-7 knockdown inhibited colony formation in these BC cell lines (Fig. 2E, F). The interference efficiency of Importin-7 knockdown was also successfully verified in these cells by immunoblotting experiments (Fig. 2G). Together, the above findings consistently confirm the significance of Importin-7 on the malignant proliferation of BC.

### Knockdown of Importin-7 induces cell cycle arrest in BC

**Fig. 3:**
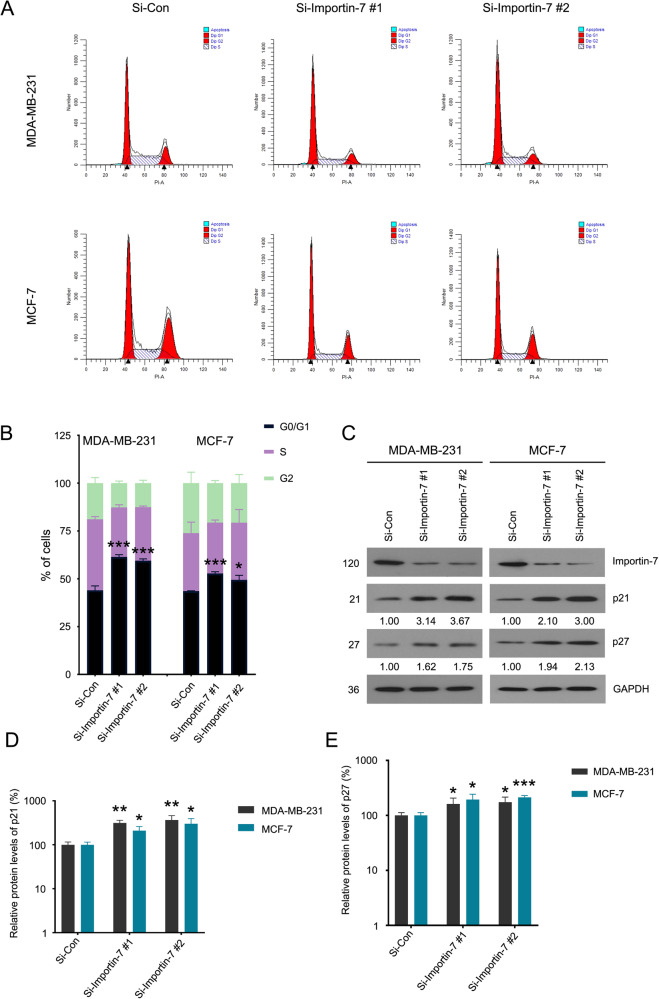
Knockdown of Importin-7 induces cell cycle arrest in BC. **A** Cell cycle distribution assay by flow cytometry was performed in MDA-MB-231 and MCF-7 cells transfected with Importin-7 or control siRNAs for 48 h. **B** Quantitative data of A are shown. Mean ± SD (*n* = 3). **C** Western blot of Importin-7, p21, and p27 in MDA-MB-231 and MCF-7 cells transfected with Importin-7 or control siRNAs for 48 h. **D**, **E** Quantitative data of **C** are shown. Mean ± SD (*n* = 3). *p < 0.05, **p < 0.01, ***p < 0.001, ****p < 0.0001 vs. each vehicle control.

The malignant proliferation ability of BC is closely related to the dysfunction of cell cycle checkpoints. Thus, we first performed flow cytometry to detect cell cycle distribution in BC cells treated with Importin-7 siRNAs. Our analysis showed that the knockdown of Importin-7 mainly induced arrest in the G0/G1 phase (Fig. 3A, B). Next, we performed an immunoblotting assay to explore the expression alteration of some potential molecules related to the cell cycle regulation in the checkpoint between the G0/G1 and S phases. We found that the reduction of Importin-7 resulted in a significant upregulation of cell cycle suppressors, such as p21 and p27 (Fig. 3C–E). Thus, we demonstrate that Importin-7 is crucial to drive the transition from G0/G1 to S phase in BC cells.

### Importin-7 binds to AR and USP22

**Fig. 4:**
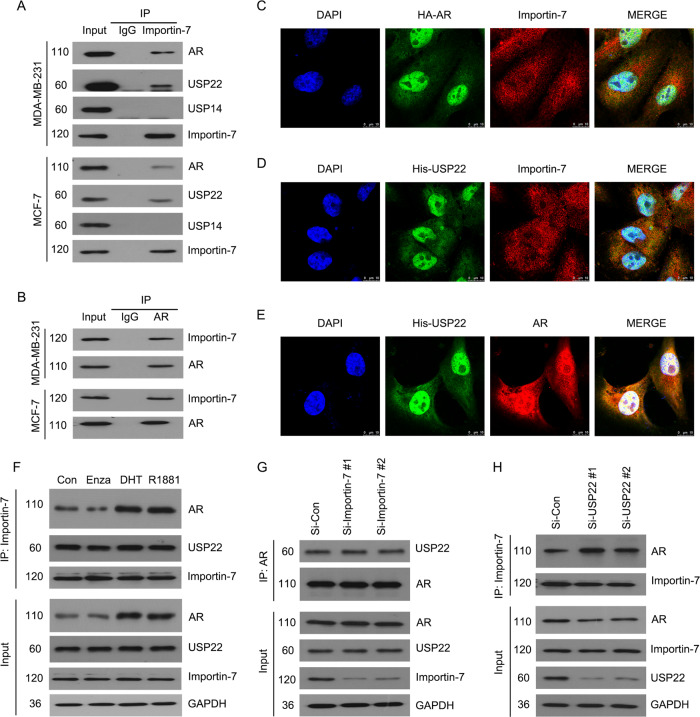
Importin-7 interacts with AR and USP22 to form a complex. **A** Co-IP assay using Importin-7 antibody and immunoblot to indicated protein molecules in MDA-MB-231 and MCF-7 cells. **B** Co-IP assay using AR antibody and immunoblot to indicate protein molecules in MDA-MB-231 and MCF-7 cells. **C** Immunofluorescence assay using HA and Importin-7 antibodies in MDA-MB-231 cells transfected with HA-AR plasmids for 48 h. Scale bars represent 10μm in shown images. **D** Immunofluorescence assay using His and Importin-7 antibodies in MDA-MB-231 cells transfected with His-USP22 plasmids for 48 h. Scale bars represent 10 μm in shown images. **E** Immunofluorescence assay using His and AR antibodies in MDA-MB-231 cells transfected with His-USP22 plasmids for 48 h. Scale bars represent 10μm in shown images. **F** Co-IP and western blot assays were performed to determine the interaction of Importin-7 with USP22 and AR in MDA-MB-453 cells androgen-starved in 10% charcoal-dextran stripped FBS (CDS–FBS) for 2 d and then exposed to enzalutamide (20 μM), DHT (10 nM) and R1881 (10 nM) for 24 h. **G** Co-IP and western blot assays were performed to determine the interaction of AR and USP22 in MDA-MB-453 cells transfected with Importin-7 or control siRNAs for 48 h. **H** Co-IP and western blot assays were performed to determine the interaction of Importin-7 and AR in MDA-MB-453 cells transfected with USP22 or control siRNAs for 48 h.

We previously conducted a series of studies on the degradation mechanism of AR and proposed that promoting the protein degradation of AR by targeting USP14 induces growth arrest and apoptosis in ER-negative BC [21, 25]. In addition, several studies have affirmed the importance of intervening AR signaling for treating ER-negative BC [16–18, 21]. AR is an essential transcription factor, which needs to be transported into the nucleus to function after being synthesized in the cytoplasm through translational folding and other processes. However, during this process, AR is at risk of being marked and degraded by the ubiquitin–proteasome system in the cytoplasm [26, 27]. The deubiquitinating enzymes USP14 and USP22 have often cited factors for AR evading the degradation fate and maintaining activity [20, 22, 24, 26, 27]. We, therefore, further explore the interaction between Importin-7 and AR/USP22/USP14 in BC by co-immunoprecipitation and immunofluorescence experiments. The results showed that Importin-7 can interact with AR and USP22 but not USP14 (Fig. 4A, B). In addition, our immunofluorescence analysis showed that Importin-7 binds to AR or USP22 in both the nucleus and cytoplasm (Fig. 4C–E), suggesting that Importin-7 may regulate the nuclear translocation of AR and USP22.

Next, we conducted a more in-depth study of the Importin-7–AR–USP22 complex by the treatment of AR inhibitor (enzalutamide) or AR ligands, including DHT and synthetic androgen R1881. We showed that the stimulation of AR ligands significantly up-regulated the expression level of AR but did not alter the expression of Importin-7 and USP22. Moreover, the expression levels of Importin-7, USP22, and AR were not affected by enzalutamide treatment. DHT and R1881 stimulation resulted in more AR binding by Importin-7, although this was more likely because DHT and R1881 induced an increase in AR, but it still showed that the level of AR carried by Importin-7 was unsaturated and variable. Enzalutamide treatment inhibited the interaction between Importin-7 and AR without changing the overall expression levels of these proteins. (Fig. 4F). Then, we further explored whether the combination of Importin-7, AR, and USP22 is competitive with each other. However, we found that the knockdown of Importin-7 did not affect the binding of AR–USP22 and the protein expression levels of AR and USP22 (Fig. 4G). It is worth noting that the knockdown of USP22, although leading to an insignificant down-regulation of AR, allows Importin-7 to bind more AR, suggesting that USP22 is possibly one of the cargoes of Importin-7 (Fig. 4H). Together, the above results indicate that Importin–7–AR–USP22 might form a relatively stable complex, even if AR and USP22 may competitively bind to Importin-7.

### Knockdown of Importin-7 restricts nuclear translocation of AR and USP22

**Fig. 5:**
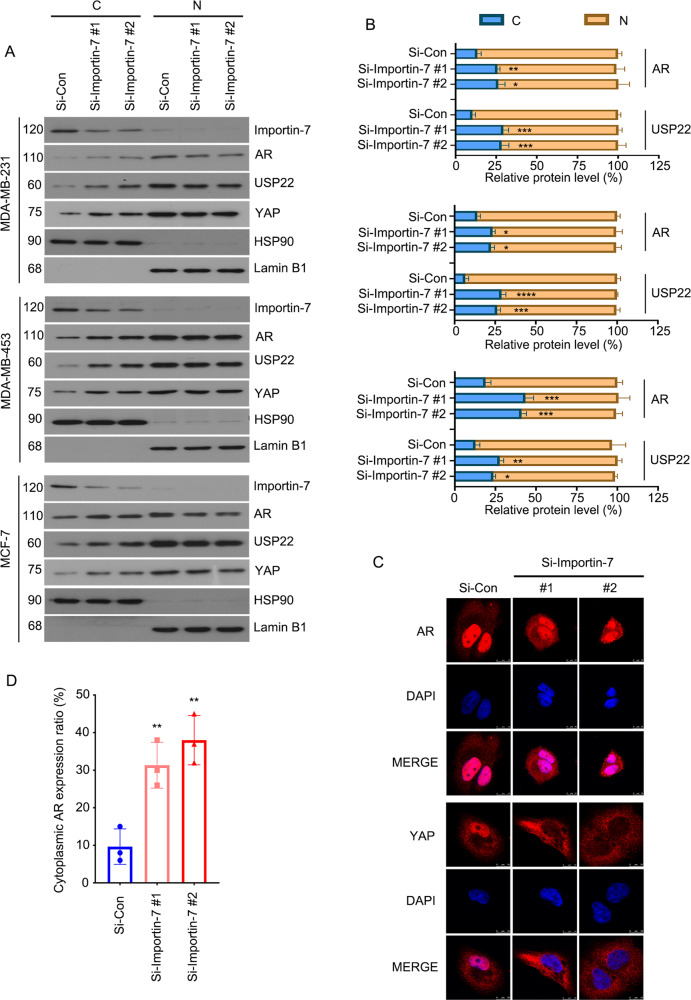
Knockdown of Importin-7 restricts nuclear translocation of AR and USP22. **A** Western blot of Importin-7, AR, and USP22 protein levels in cytoplasmic and nuclear of MDA-MB-231, MDA-MB-453, and MCF-7 cells transfected with Importin-7 or control siRNAs for 48 h. **B** Quantitative data of AR and USP22 protein levels are shown. Mean ± SD (*n* = 3). **C** Immunofluorescence assay using AR and YAP antibodies in MDA-MB-231 cells transfected with Importin-7 or control siRNAs for 48 h. Scale bars represent 10 μm in shown images. **D** Quantitative data of **C** are shown. Mean ± SD (*n* = 3). *p < 0.05, **p < 0.01, ***p < 0.001, ****p < 0.0001 vs. each vehicle control.

In order to address whether Importin-7 mediates the nuclear translocation of AR and USP22, cytoplasmic and nuclear proteins were isolated in BC cells treated with Importin-7 siRNAs or control sequences. Immunoblotting analysis was subsequently conducted to detect the expression of Importin-7, USP22, and AR in the nucleus and cytoplasm, respectively. YAP, known as an Importin-7 cargo [10], was used as a positive control. We showed that the downregulation of Importin-7 causes AR and USP22 proteins to be retained in the cytoplasm and reduced in the nucleus (Fig. 5A, B). Besides, the results of the immunofluorescence assay also support the above findings (Fig. 5C, D). These data demonstrate that the knockdown of Importin-7 restricts the nuclear translocation of AR and USP22.

### Silence of Importin-7 enhances the sensitivity of BC cells to enzalutamide

As we have demonstrated previously, increasing the responsiveness of BC to AR signaling suppression with enzalutamide by inhibition of USP14-induced AR degradation was elaborated as a potential therapeutic strategy [21, 25]. Here, to further investigate whether the arrest of the nuclear translocation of AR–USP22 complex triggered by the knockdown of Importin-7 could enhance the sensitivity of BC to enzalutamide.

**Fig. 6:**
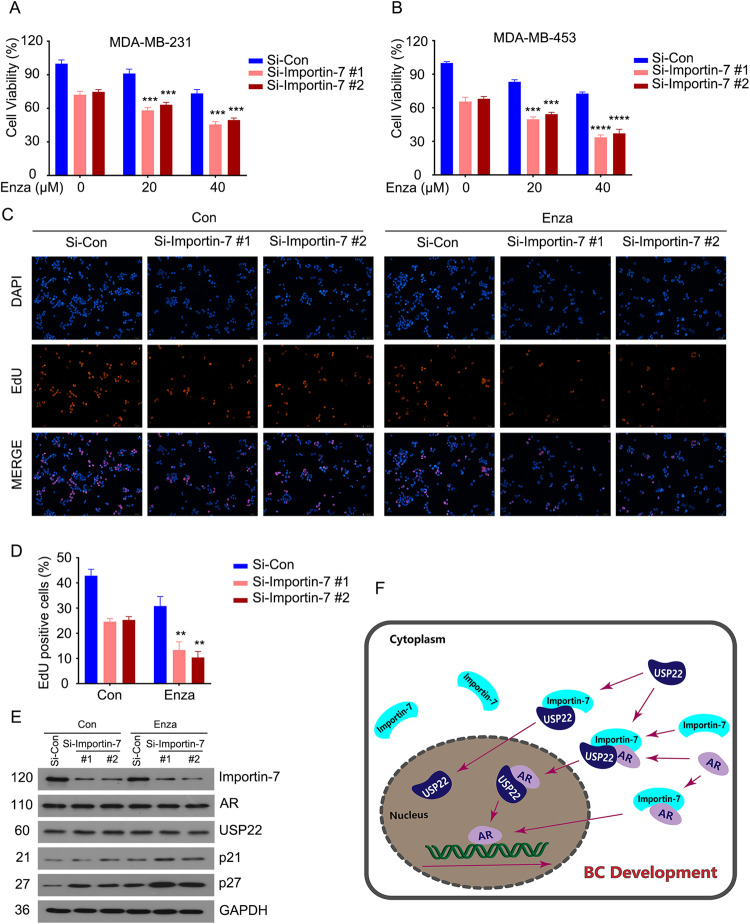
Restricting the expression level of Importin-7 enhances the sensitivity of BC to enzalutamide. **A**, **B** CCK8 assays of MDA-MB-231 and MDA-MB-453 cells transfected with two pairs of Importin-7 or control siRNAs for 12 h, then treated with or without enzalutamide for 36 h. **C** EdU staining assay was performed in MDA-MB-453 cells transfected with Importin-7 or control siRNAs for 12 h, then treated with or without enzalutamide (20 μM) for 36 h. **D** Quantitative data of **C** are shown. Mean ± SD (*n* = 3). **E** Western blot of Importin-7, AR, USP22, p21, and p27 in MDA-MB-453 cells transfected with Importin-7 or control siRNAs for 12 h, then treated with or without enzalutamide (20 μM) for 36 h. **F** A proposed model of Importin-7-induced nuclear translocation of AR–USP22 to promote malignant proliferation of AR-positive breast cancer. **p < 0.01, ***p < 0.001, ****p < 0.0001 vs. each vehicle control.

We first confirmed that the knockdown of Importin-7 could enhance the growth inhibition of cancer cells by enzalutamide using CCK8 assay (Fig. 6A, B) and EdU staining assay (Fig. 6C, D). Further exploration of the mechanism revealed that the knockdown of Importin-7 also amplified enzalutamide-induced upregulation of cell cycle repressors (Fig. 6E). Thus, these findings further confirm that Importin-7 may be developed as a valuable target for treating BC.

## Discussion

BC, the greatest threat to women’s life and health, is complex cancer that exhibits high heterogeneity between and even within tumors [1, 2, 28, 29]. In addition to traditional classifications, an increasing number of ER-negative and triple-negative BC subtypes have been identified [2, 3, 29]. In recent years, studies have also pointed out that multiple BC subtypes can coexist in tumors and that even breast tumors may be composed of a mixture of mutually transformed BC subtypes [2, 3, 29, 30]. This means that traditional concepts may no longer be sufficient to adequately describe and treat BC. It is crucial and urgent to discover new potential therapeutic targets and develop new therapeutic strategies. Therefore, we performed biological mass spectrometry detection on the collected benign breast hyperplasia tissues and invasive BC tissues and analyzed the differences in protein expression levels. Based on the current in vitro screening, we discovered that Importin-7 is essential for sustaining the proliferative ability of BC.

In order to ensure the normal progress of various life processes of cells, the subcellular spatial distribution of proteins needs to be precisely regulated, which depends on the selective and efficient transport of substrate proteins by a series of transport receptors [5, 9]. Dysregulation of nucleocytoplasmic transport function in cancer may lead to aberrant cell growth signaling and inactivation of apoptotic pathways, which are critical for tumor growth and progression [7, 31]. Importin-7, a member of the nuclear transporter family, mediates the entry of key biological molecules, including ribosomal protein, histone H1, glucocorticoid receptor, etc., into the nucleus [11, 32, 33]. Increasing investigations have shown that Importin-7 universally exists in various cancers [12–14]. In this study, we verified its high expression status in BC and explored its importance to the malignant proliferation of BC cells. Mechanistically, the knockdown of Importin-7 induces G0/G1 arrest of the cell cycle by upregulating the expression of cycle repressors, including p21 and p27, thereby suppressing cancer growth and malignant progression.

Next, we discovered that Importin-7 could physically interact with AR and regulate its nuclear translocation in BC cells. Previously, we have confirmed the importance of AR in the progression of ER-negative BC [21, 25]. In addition, multiple studies have collectively indicated that AR inhibition in various ways can benefit the treatment of ER-negative BC [16–18, 21]. Therefore, inhibition of the nuclear translocation of AR by targeting Importin-7 is a potential therapeutic strategy. It is well known that the proteasome regulates the stability of proteins in cells depending on the dynamic balance of ubiquitination and deubiquitination levels. In AR-positive BC, the maintenance of AR expression status and functional activity drives malignant proliferation, implicating that there is a mechanism that prevents AR from being degraded before entering the nucleus to function. We, therefore, have a strong interest in AR’s deubiquitinases that interact with Importin-7. USP14 and USP22 are two frequently mentioned and well-defined AR deubiquitinating enzymes. Interestingly, we found that USP22, one of the maintainers of AR [22–24], whose nuclear translocation is also regulated by Importin-7, although knockdown of Importin-7 may not affect the formation of the AR–USP22 complex.

In addition, we also observed that AR expression levels were upregulated after DHT and R1881 stimulation, and Importin-7-bound AR was also up-regulated to an almost equal extent, indicating that the level of AR carried by Importin-7 was not saturated. These results suggested that the efficiency of Importin-7 in AR delivery may be enhanced by androgen stimulation. Additionally, we also found that silencing USP22 resulted in a less pronounced downregulation of AR. Interestingly, silencing USP22 increased the level of Importin-7 binding to AR, implying a competing binding mechanism, which further supports the view that USP22 is also individually one of the cargos of Importin-7.

Furthermore, we further explored and found that the knockdown of Importin-7 enhanced the sensitivity of BC to enzalutamide. Knockdown of Importin-7 synergizes with enzalutamide to induce cycle arrest and promote cancer cell growth inhibition. These results suggest that the identification and development of Importin-7 inhibitors may be promising for clinical application.

## Conclusion

Overall, through the analysis of clinical samples combined with experimental studies of cellular and molecular biology, the current study identifies a molecule key to BC progression, Importin-7. In this study, we focused on one of its mechanisms of action: forming a protein complex with USP22 and AR and promoting the nuclear transport of AR–USP22. In addition, this study also provides a rationale for a therapeutic strategy to limit the malignant progression of AR-positive BC by inhibiting the high expression state of Importin-7.

## Materials and methods

### BC samples

Fresh tissues were kindly donated from patients with BC or benign breast hyperplasia. All samples were obtained from the discarded material utilized for routine laboratory tests at the Department of Breast Surgery, First People’s Hospital of Foshan (Foshan, China). This study was performed with the approval of the Medical Ethics Committee of the First People’s Hospital of Foshan.

### LC–MS/MS analysis in DIA mode

The fresh benign breast hyperplasia and malignant BC tissues were stored in dry ice, and subsequent quantitative liquid chromatography–tandem mass spectrometry (LC–MS/MS) via data-independent acquisition (DIA) [34, 35] was performed in Wininnovate Bio (Shenzhen, China).

### Cell lines, chemicals, and antibodies

BC cell lines were obtained from ATCC. Cell culture conditions are listed in Table S1. Chemicals are listed in Table S2. Antibodies are listed in Table S3.

### Cell proliferation assays

Cell viability was detected by using a cell counting kit-8 (CCK8) assay (Dojindo Molecular Technologies, Japan), as we have described in the past [24]. Cells were seeded in a 96-well plate for 24 h with a complete medium containing 2000 cells per well. After treatment, CCK8 reagent was added to cells and further cultured for 3 h in the dark. Then according to the instructions of CCK8, the cells were measured with a microplate reader at a wavelength of 450 nm from three independent experiments.

The EdU staining assay (RiboBio, Guangzhou, China) was used to detect the DNA replication rate of BC cells. The experimental operation methods and steps were carried out strictly in accordance with the kit instructions and the way we reported in the past [36].

For the colony formation experiment, cells were reseeded in a 6-well plate for two weeks after being treated as directed. The cells were washed with PBS twice, fixed for 15 min with 4% paraformaldehyde, then stained for 5 min with crystal violet solution. Finally, from three separate studies, the number of colonies larger than 60 mm was counted.

### Flow cytometry analysis

Consistent with the previous description, cell cycle distribution, and apoptosis were detected by flow cytometry [24, 36, 37]. For cell cycle assay, after transfected with Importin-7 siRNAs, cells were digested and washed twice with 4 °C PBS and fixed with a mixture containing 2 ml cold 70% ethanol solution and 500 μl PBS at 4 °C overnight. After being washed with PBS, cells were prepared into a single cell suspension using a Propidium Iodide (PI)-containing cell cycle detection kit (KGA512, KeyGEN BioTECH, Nanjing, China) according to the instruction and left to stand in the dark for 30 min. Three independent tests for cell cycle distributions of each group were ultimately examined with flow cytometry.

For apoptosis assay, cells were treated according to the indicated conditions, then collected and washed twice using 4 °C PBS. An Annexin V-FITC/PI-containing apoptosis detection kit (KGA108, KeyGEN BioTECH, Nanjing, China) was used to stain the apoptotic cells according to the instruction. Flow cytometry was performed to determine the apoptotic rate from three independent experiments.

### siRNA/shRNA and plasmid transfection

The RNA interfering and plasmid transfection assays were performed in BC cells and conducted as previously reported [24, 36, 38]. The siRNAs and shRNAs were purchased from RiboBio and GeneChem (Shanghai, China), respectively. The siRNA and shRNA sequences are listed in Table S4.

For siRNAs and plasmids transfection, the transfection mixture was prepared with siRNAs or plasmids, RPMI opti-MEM (Gibco), and TransIT-X2® Dynamic Delivery System (MIR 6000, Mirus Bio) according to the system in the User Guide of MIR 6000. The transfection mixture was then added to plates where cells had been seeded for 24 h.

For lentivirus transfection, lentivirus containing shRNAs targeting Importin-7 or control shRNAs were generated. Firstly, a complete medium containing polybrene (5 μg/ml) was used to incubate the BC cells for 15 min, and lentivirus was then added into plates for 48 h. Medium containing puromycin (2 μg/ml) was used to eliminate the unsuccessfully infected cells.

### Immunofluorescence and immunohistochemistry assays

An immunofluorescence experiment was carried out as previously described [24, 36, 38]. In short, cells were seeded in a glass bottom petri dish and treated as the figure legends. Cells were washed with 4 °C PBS and then fixed with 4% paraformaldehyde for 30 min. After fixation, cells were permeabilized with 0.1% Triton X-100 and blocked with 5% BSA for 30 min. Primary antibodies were added and incubated the cells overnight in the cold. Cells were then incubated with the secondary antibodies in the dark for 1 h. After the addition of the DAPI solution, a confocal microscope (Leica TCS SP8) was used to preserve fluorescence. Immunohistochemistry (IHC) experiment was also carried out as in our previous reports [24, 36]. The samples of tumor were immunohistochemically stained with a MaxVision kit (Mixin Biotech), according to the kit and antibody instructions. Then, the slides were counterstained with hematoxylin, and the primary antibodies were detected by the DAB method. The optical microscope (Leica DM2500) was used to observe and photograph.

### Extraction of nuclear and cytoplasmic proteins

Separation of proteins between the nucleus and cytoplasm was strictly performed in accordance with the user guide for Nuclear and Cytoplasmic Protein Extraction Kits (KGP150, KeyGEN BioTECH, Nanjing, China).

### Western blotting and co-IP assays

Protein samples were collected at low temperatures using the cell lysis buffer (Cell Lysis Buffer (#9803) (Cell Signaling Technology, Beverly, MA, USA), protease inhibitor mixture, phosphatase inhibitor mixture, and PMSF (Keygen, Nanjing, Jiangsu, China). The western blotting and co-immunoprecipitation (co-IP) experiments, including the preparation steps of the protein samples, were completely consistent with our previous descriptions and reports [21, 24, 36, 38].

### Statistical analysis

Experimental results are reported as the mean S.D. of three separate experiments. When determining statistical probability, the paired/unpaired Student’s *t*-test or one-way ANOVA is utilized. GraphPad Prism 9 and SPSS 16.0 software were utilized for statistical analysis. A *p* value < 0.05 was considered statistically significant. ^*^p < 0.05, ^**^p < 0.01, ^***^p < 0.001, ^****^p < 0.0001 vs. each vehicle control.

## Supplementary information

Supplementary Tables

Original Data File

## Data Availability

Data are available from the corresponding author upon reasonable request.
