# Supplementary material for: Nuclear transport maintenance of USP22-AR by Importin-7 promotes breast cancer progression
Source: Cell Death Discov. 2023 Jul 1;9:211. doi: 10.1038/s41420-023-01525-8 (PMC10313651; doi:10.1038/s41420-023-01525-8)
Supplement: Supplementary file 1 — Supplementary Tables [file 41420_2023_1525_MOESM1_ESM.pdf]

Table S1. Information of cell culture used in the study.

Cell line	Cell line supplier	Culture condition	Medium	Medium manufacturer	FBS manufacturer
MDA-MB-231	ATCC	37 °C 5% CO_2	DMEM Hams F-12 50/50 Mix with 10% FBS	Corning	BioInd
MCF-7					
MDA-MB-453			DMEM with 10% FBS		

Table S2. Information of antibodies used in the study.

Antibodies	Manufacturer	Catalog number	Application	Dilution
anti-Importin7	Abcam	#ab99273	WB	1:1000
			IP	1:50
			IF	1:400
			IHC	1:100
anti-GAPDH	Cell Signaling Technology	#5174	WB	1:1000
anti-p21	Cell Signaling Technology	#2947	WB	1:1000
anti-p27	Cell Signaling Technology	#3686	WB	1:1000
anti-IgG	Cell Signaling Technology	#3900	IP	1:50
anti-AR	Cell Signaling Technology	#14793	WB	1:1000
			IP	1:50
			IF	1:400
anti-USP22	Abcam	# ab195289	WB	1:1000
			IP	1:50
anti-USP14	Cell Signaling Technology	#11931	WB	1:1000
anti-YAP	Cell Signaling Technology	#14074	WB	1:1000
			IF	1:200

anti-HSP90	Cell Signaling Technology	#4877	WB	1:1000
anti-Lamin B1	Cell Signaling Technology	#13435	WB	1:1000
anti-His-Tag	Abcam	#ab5000	IF	1:200
anti-HA-tag	Cell Signaling Technology	#2367	IF	1:200
anti-rabbit IgG (HRP Conjugate)	Cell Signaling Technology	#7074	WB	1:5000
anti-mouse IgG (HRP Conjugate)	Cell Signaling Technology	#7076	WB	1:5000
Mouse Anti-rabbit IgG (HRP Conjugate)	Cell Signaling Technology	#5127	WB	1:2000
Goat Anti-Mouse IgG H&L (Alexa Fluor® 488)	Abcam	#ab150113	IF	1:200
Goat Anti-Rabbit IgG H&L (Alexa Fluor® 647)	Abcam	# ab150079	IF	1:200

Table S3. Information of chemicals used in the study.

**Chemicals**	**Manufacturer**	**Catalog number**
Enzalutamide	Selleck	#S1250
Dihydrotestosterone	Selleck	#S4757
R1881	AbMole	#M8128

Table S4. The target sequences of si/sh-RNAs used in the study.

**siRNA**	**Target sequence**
Importin7 si/sh-RNA #1	5′- GCAAGAAGACCCTTACGAA -3′
Importin7 si/sh-RNA #2	5′- GATGGAGCCCTGCATATGA -3′
USP22 si-RNA #1	5′-GCAGCGAAAAGCTTGGAAA -3′
USP22 si-RNA #2	5′-GTACGGAGGCATCTACTGT -3′
