# Supplementary material for: Nuclear transport maintenance of USP22-AR by Importin-7 promotes breast cancer progression
Source: Cell Death Discov. 2023 Jul 1;9:211. doi: 10.1038/s41420-023-01525-8 (PMC10313651; doi:10.1038/s41420-023-01525-8)
Supplement: Supplementary file 2 — Original Data File [file 41420_2023_1525_MOESM2_ESM.pdf]

Figure.1F

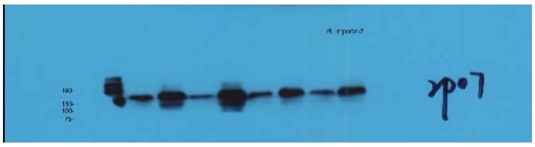

Importin-7 （P#1-4）

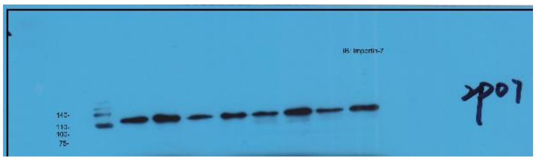

Importin-7 （P#5-8）

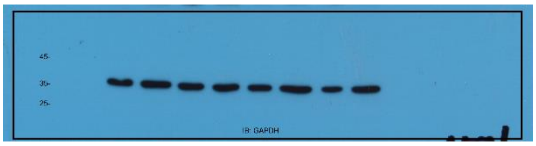

GAPDH （P#1-4）

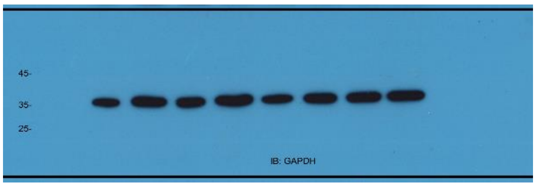

GAPDH （P#5-8）

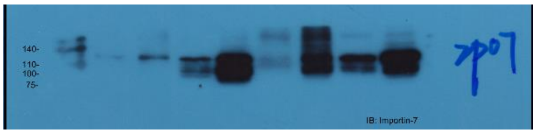

Importin-7 （P#9-12）

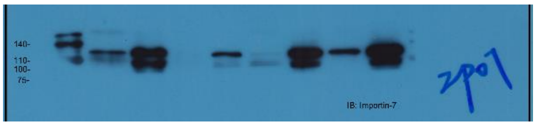

Importin-7 （P#13-16）

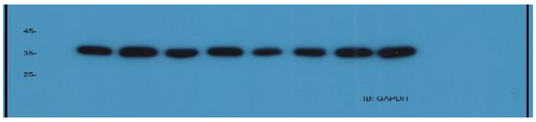

GAPDH （P#9-12）

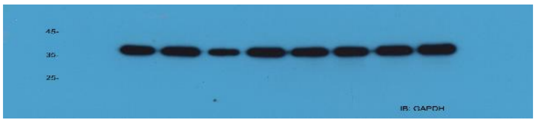

GAPDH （P#13-16）

Figure.2B

MDA-MB-231

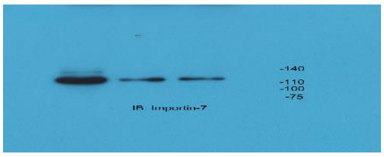

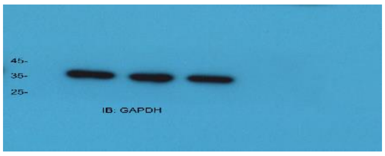

Importin-7 GAPDH

MDA-MB-453

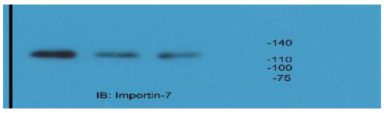

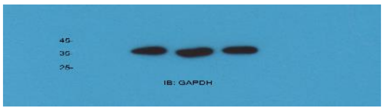

Importin-7 GAPDH

MCF-7

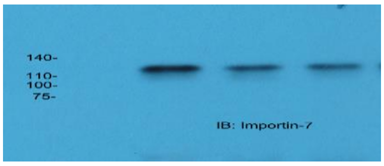

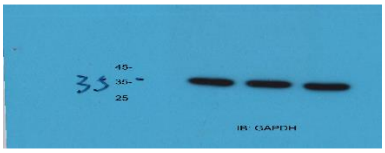

Importin-7 GAPDH

Figure.2G

MDA-MB-453

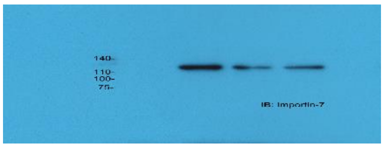

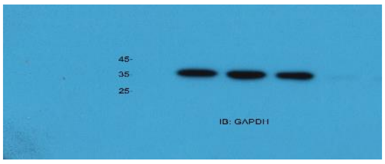

Importin-7 GAPDH

MCF-7

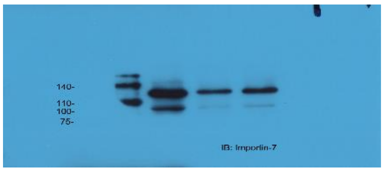

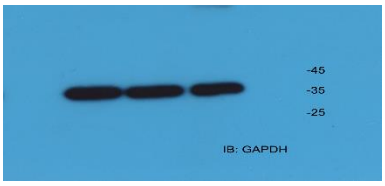

Importin-7 GAPDH

Figure.3C

MDA-MB-231

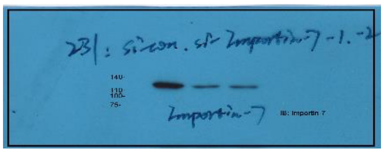

Importin-7

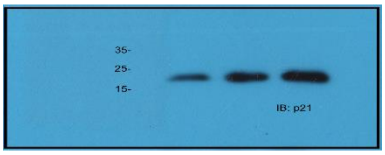

p21

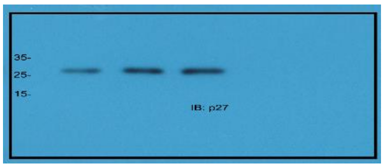

p27

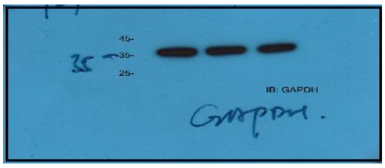

GAPDH

MCF-7

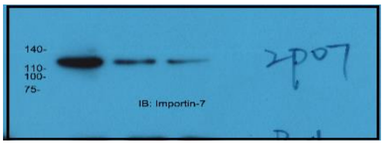

Importin-7

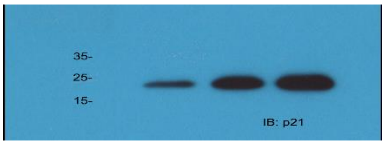

p21

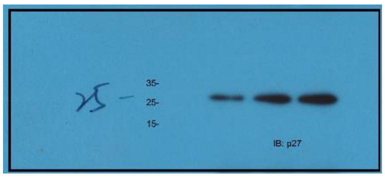

p27

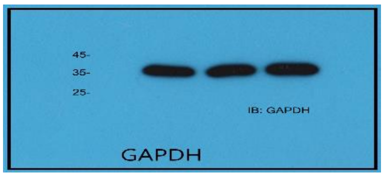

GAPDH

Figure.4A

MDA-MB-231

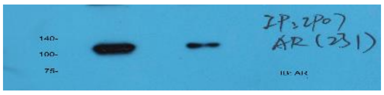

AR

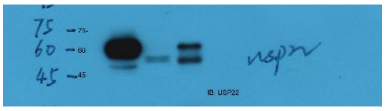

USP22

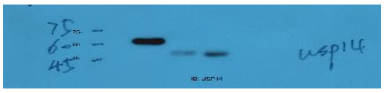

USP14

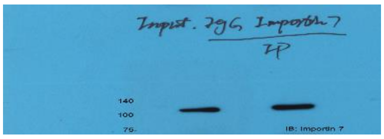

Importin-7

MCF-7

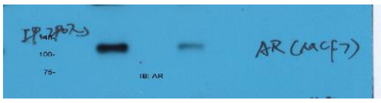

AR

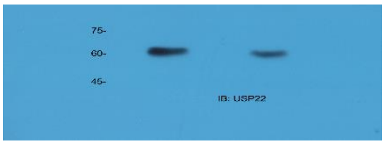

USP22

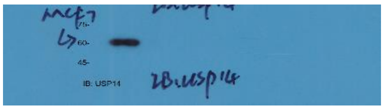

USP14

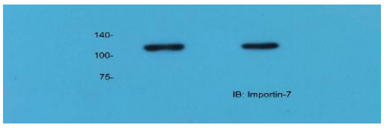

Importin-7

Figure.4B

MDA-MB-231

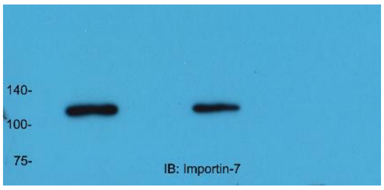

Importin-7

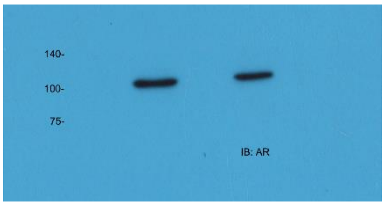

AR

MCF-7

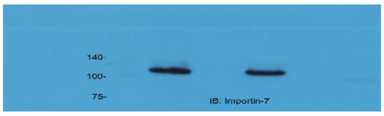

Importin-7

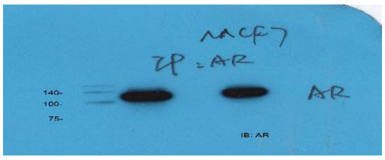

AR

Figure.4F

IP: Importin-7

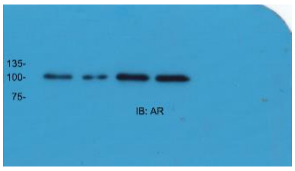

AR

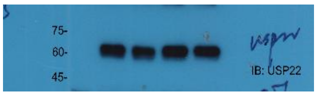

USP22

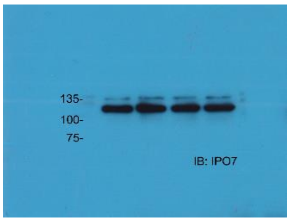

Importin-7

Input

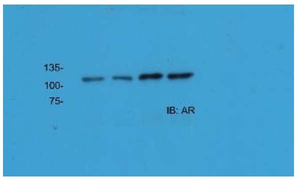

AR

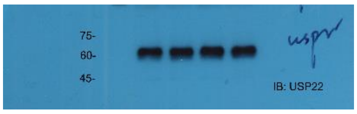

USP22

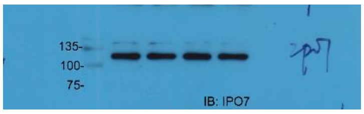

Importin-7

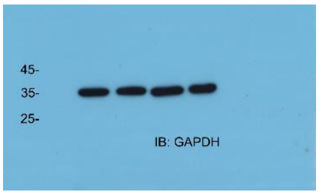

GAPDH

Figure.4G

IP: AR

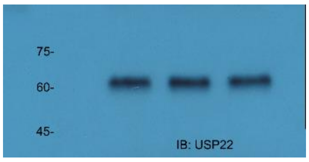

USP22

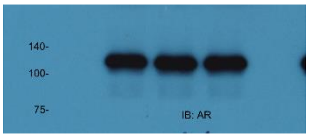

AR

Input

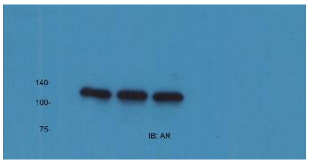

AR

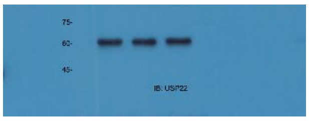

USP22

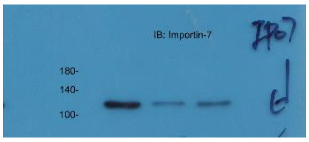

Importin-7

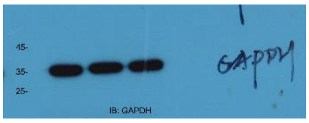

GAPDH

Figure.4H

IP: Importin-7

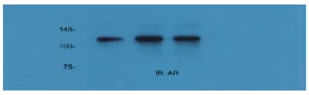

AR

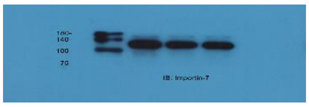

Importin-7

Input

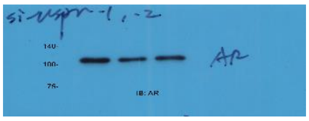

AR

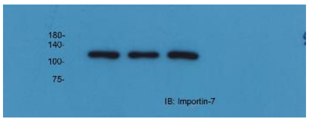

Importin-7

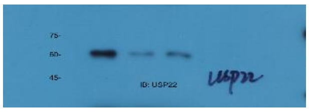

USP22

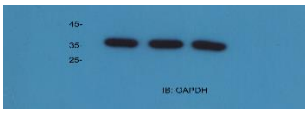

GAPDH

Figure.5A

MDA-MB-231

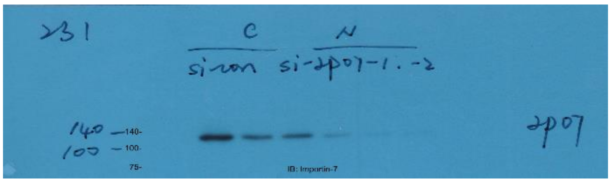

Importin-7

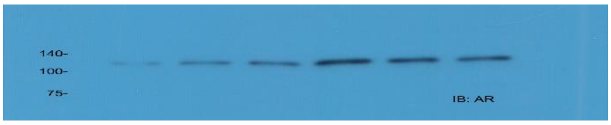

AR

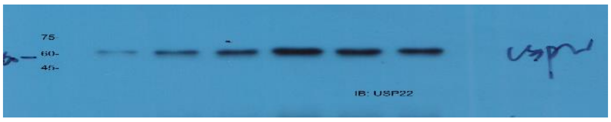

USP22

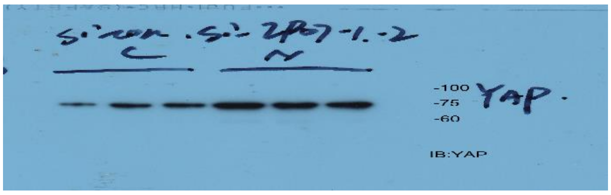

YAP

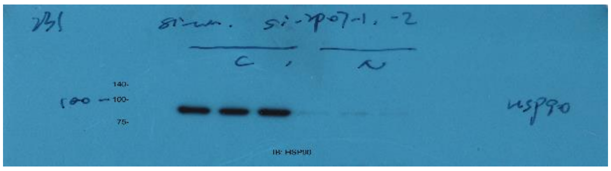

HSP90

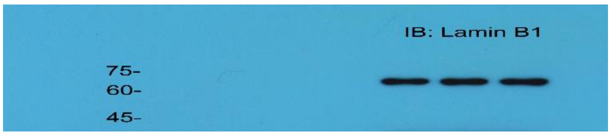

Lamin B1

MDA-MB-453

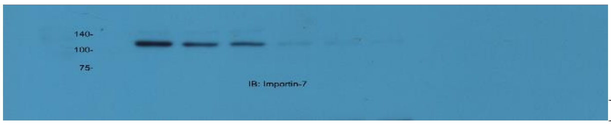

Importin-7

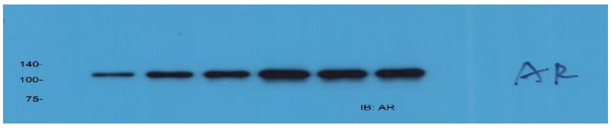

AR

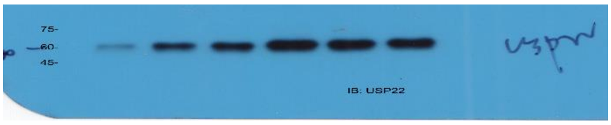

USP22

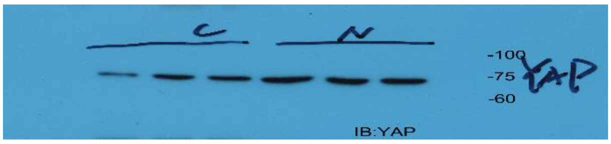

YAP

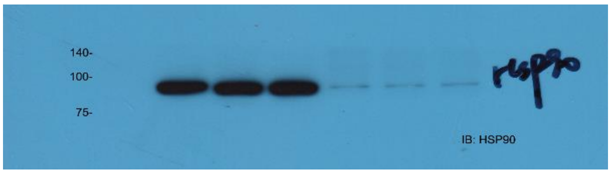

HSP90

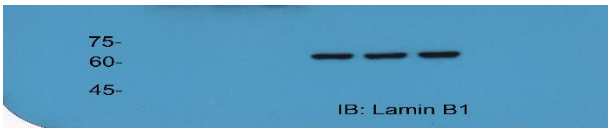

Lamin B1

MCF-7

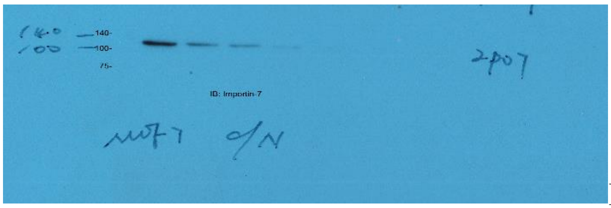

Importin-7

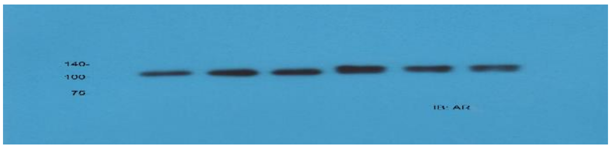

AR

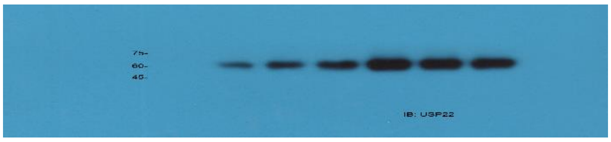

USP22

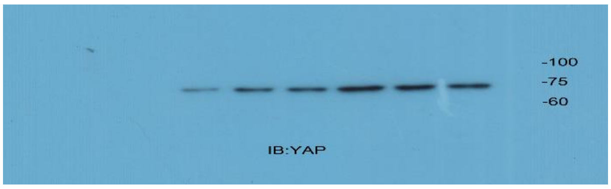

YAP

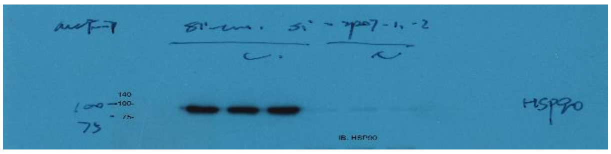

HSP90

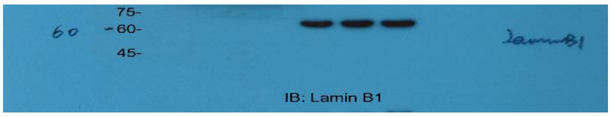

Lamin B1

Figure.6E

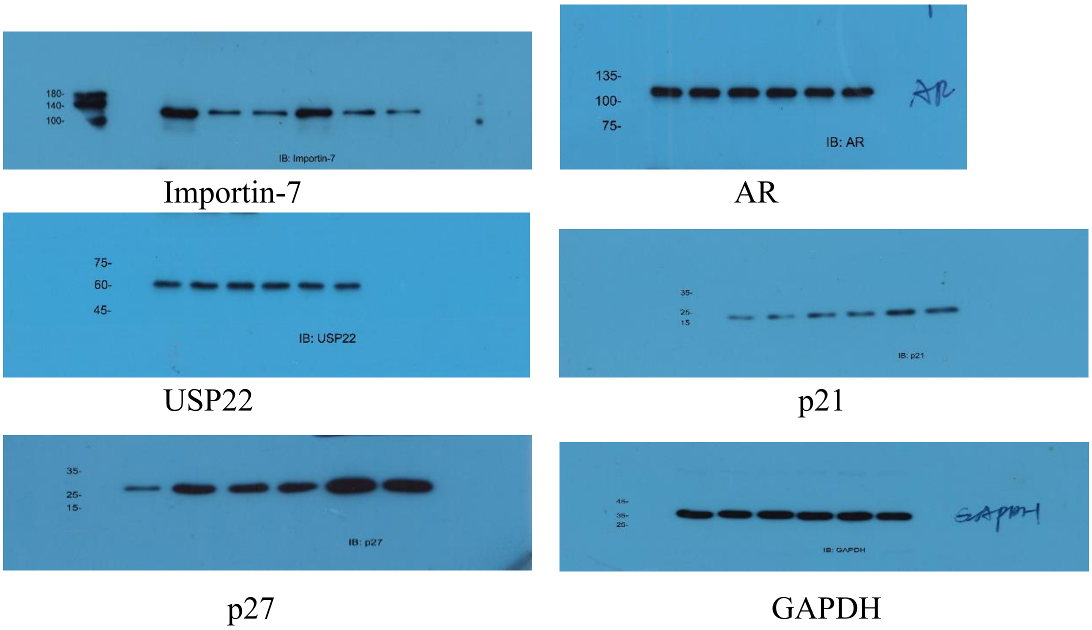
180-
140-
100-
IB: Importin-7
Importin-7
135-
100-
75-
IB: AR
AR
75-
60-
45-
IB: USP22
USP22
35-
25-
15
IB: p21
p21
35-
25-
15-
IB: p27
p27
IB: GAPDH
GAPDH
